# Glucose screening within six months postpartum among Chinese mothers with a history of gestational diabetes mellitus: a prospective cohort study

**DOI:** 10.1186/s12884-019-2276-9

**Published:** 2019-04-18

**Authors:** Zhu-yun Liu, Juan-juan Zhao, Ling-ling Gao, Alex Y. Wang

**Affiliations:** 1 Guangdong Provincial Hospital of Chinese Medicine, The Second Affiliated Hospital of Guangzhou University of Chinese Medicine, Guangzhou, China; 2 School of Nursing, Sun Yat-sen University, 74#, Zhongshan Road II, Guangzhou, 510089 China; 3 Faculty of Health, University of Technology Sydney, Sydney, New South Wales Australia

**Keywords:** Diabetes, Gestational, Health beliefs, Postpartum glucose screening

## Abstract

**Background:**

Gestational diabetes mellitus (GDM) is a risk factor for diabetes mellitus. The 75-g, 2-h oral glucose tolerance test is recommended for mothers with a history of GDM to screen for diabetes in the postnatal period. The aim of this study was to investigate the rate of glucose screening within 6 months postpartum among Chinese mothers with a history of GDM, and to identify its predictors.

**Methods:**

A prospective cohort study was conducted in a regional teaching hospital in Guangzhou, China, between July 2016 and June 2017. The participants were Chinese mothers (*n* = 237) who were diagnosed with GDM, were aged 18 years or older with no serious physical or mental disease and had not been diagnosed with type 1 or type 2 diabetes prior to their pregnancy. The revised Chinese version of the Champion’s Health Belief Model Scale and social-demographic and perinatal characteristics factors were collected and used to predict postpartum glucose screening (yes or no). Adjust odds ratio (AOR) and 95% confidence interval (95% CI) were calculated.

**Results:**

The mean age of the 237 mothers was 32.70 years (range from 22 to 44). Almost half of the mothers (45.6%) were college graduates or higher. Chinese mothers reported a high level of perceived benefits, self-efficacy, and health motivation towards postpartum glucose screening, with a mean score above 3.5.

Chinese mothers were more likely to undertake postpartum glucose screening if they were a first-time mother [AOR 2.618 (95% CI: 1.398–4.901)], had a high perceived susceptibility score [AOR 2.173 (95% CI: 1.076–4.389)], a high perceived seriousness score [AOR 1.988 (95%CI: 1.020–3.875)] and high perceived benefits score [AOR 2.978 (95%CI: 1.540–5.759)].

**Conclusion:**

The results of this study will lead to better identification of mothers with a history of GDM who may not screen for postpartum glucose abnormality. Health care professionals should be cognizant of issues that may affect postpartum glucose screening among mothers with a history of GDM, including parity, perceived susceptibility, perceived seriousness and perceived benefits.

**Electronic supplementary material:**

The online version of this article (10.1186/s12884-019-2276-9) contains supplementary material, which is available to authorized users.

## Background

Diabetes mellitus is a complex chronic condition with serious physical, psychological, and clinical complications for the individuals affected [1]. Gestational diabetes mellitus (GDM) is a risk factor for diabetes mellitus. Mothers with a history of GDM are 7 times more likely to develop type 2 diabetes mellitus later in life than those without a history of GDM [2]. In fact, up to 70% of mothers with a history of GDM will develop type 2 diabetes mellitus if there is no intervention [3]. In mainland China, GDM has become one of the most common complications during pregnancy. A recent survey of 15,194 Chinese pregnant women found that 19.7% of them were diagnosed with GDM, which was higher than the average morbidity worldwide [4].

Given the impact of prolonged undetected hyperglycaemia, prevention and early diagnosis of diabetes is cost-effective and important for public health [5–7]. It is recommended that all mothers with a history of GDM should screen for glucose in the postpartum period [8, 9]. This can help to detect glucose abnormality and then provide early preventive interventions. Many mothers diagnosed with GDM however, do not undergo blood glucose screening within the postnatal period [10–12]. One report indicated that only 13.1% Chinese mothers (282 out of 2152) with a history of GDM were screened for blood glucose in the postnatal period [10].

A number of international studies have suggested that screening behaviour can be predicted by health beliefs [13–15]. According to the Health Belief Model, health beliefs refer to subjective feelings and cognition when forming healthy behaviours. The Health Belief Model consists of six constructs: perceived susceptibility, perceived seriousness, perceived benefits, perceived barriers, cues to action and self-efficacy [16]. The Health Belief Model proposes that people must believe that, even in the absence of any symptom, the disease may exist. When people find themselves at risk of the disease (perceived susceptibility), realize that the disease has serious potential consequences (perceived seriousness), believe that barriers of that behaviour (perceived barriers) are less than the obtained benefits (perceived benefits), and believe that they are able to undertake health behaviour activities (self-efficacy), they are more likely to accomplish screening behaviour [17].

Apart from health beliefs, the literature shows that other factors are associated with postpartum glucose screening. These include socio-demographic characteristics such as age, race, parity, income, education, pre-pregnancy weight or body mass index (BMI) [18–20] and perinatal characteristics which include insulin use during pregnancy, medication use during pregnancy, postpartum visits, and gestational weight gain [11, 19, 21].

It is important to identify a set of factors associated with postpartum glucose screening in mothers with a history of GDM. Once identified, effective counselling and promotion policies can be therefore implemented to improve rates of postpartum glucose screening. However, limited evidence is available on the association between postpartum glucose screening and health beliefs and other factors among mothers with a history of GDM. This study aims to investigate the rate of the postpartum glucose screening and identify its predictors among Chinese mothers with a history of GDM.

## Methods

### Study design

A prospective cohort study was conducted.

### Settings and participants

The study was carried out in Guangzhou, the capital of Guangdong Province located in southeast China. Guangzhou is classed as a first-tier city with a population of around 16 million. More than half of the employed women have received a tertiary education with 82.3% having a higher qualification in some Districts of Guangzhou [22].

One leading hospital in Guangzhou was selected for this study. The incidence rate of GDM in the study hospital was 17% from 2013 to 2016, which is similar to the average incidence of GDM in China in 2010 which was 17.5% [23]. The participating mothers were recruited from 2106 mothers who gave birth between July and December 2016.

GDM is diagnosed based on the criteria of the International Association of Diabetes and Pregnancy Study Group (IADPSG), recommended by the Chinese Medical Association [9]. The IADPSG criteria define GDM as a fasting blood glucose (FPG) > 5.1 mmol/l, a 1-h glucose level > 10.0 mmol/l or a 2-h glucose level > 8.5 mmol/l. [9]. The mothers were recruited from the postnatal wards of the study hospital. Mothers were eligible if they were diagnosed with GDM, aged 18 years or older, had given birth to a term (> 36 complete weeks of gestation) singleton, with no serious physical or mental disease and had not been diagnosed with type 1 or type 2 diabetes prior to their pregnancy. Mothers were excluded if they had multiple deliveries, had serious physical or mental health conditions, had re-conceived within 6 months postpartum or were diagnosed with type 1or type 2 diabetes mellitus prior to pregnancy.

### Study factors

A revised Chinese version of the Champion’s Health Belief Model Scale (RC-CHBMS) was used to measure health beliefs. The original Chinese version of the Champion’s Health Belief Model Scale was a scale adapted from Champion [24, 25] to measure beliefs related to liver cancer screening [26]. It was subsequently revised to measure beliefs related to diabetes and postpartum screening for diabetes. The RC-CHBMS has been validated in Chinese women with a history of GDM. Reported internal consistency for the scale was 0.833; the six subscales’ Cronbach’s alpha coefficients ranged from 0.773 (motivation) to 0.806 (perceived benefits) [27].

The RC-CHBMS is a 33-item instrument consisting of 6 subscales: perceived susceptibility (5 items), perceived seriousness (6 items), perceived benefits (6 items), perceived barriers (6 items), health motivation (5 items) and self-efficacy (5 items). Each item was rated on a five-point Likert scale ranging from “strongly disagree” (1 point) to “strongly agree” (5 points). A higher ranking on the Likert scale indicates a stronger agreement with the health beliefs (for example, a higher benefit score indicated perception of greater benefits and a higher barrier score indicated a greater perception of barriers). All subscales were positively related to postpartum screening for diabetes, except for barriers which were negatively associated.

A self-designed social-demographic data sheet was used to collect data on maternal age, marital status, education, employment and family income, family history of diabetes and pre-pregnancy body mass index (BMI). A number of perinatal characteristics were collected, including parity, gestational age at the diagnosis of GDM, plasma glucose of OGTT during pregnancy, insulin therapy during pregnancy, gestational weight gain, glucose control during pregnancy, mode of delivery, infant feeding method and childcare assistance.

After giving informed written consent, mothers were asked to complete the RC-CHBMS and self-designed social-demographic data sheet. Both RC-CHBMS and social-demographic data sheet were collected within 4 days following the birth. Data on perinatal characteristics were collected from the mothers’ medical record.

In mainland China, the recommended ranges for blood glucose control for pregnant women with GDM are as follows: pre-prandial and fasting plasma glucose between 3.3 and 5.6 mmol/l (60–99 mg/dl) and a 2-h post-prandial plasma glucose between 5.6 and 7.1 mmol/l (100–129 mg/dl). At each prenatal visit, the obstetrician checks the self-monitoring record of every pregnant women with GDM. If more than 70% of the self-monitored blood glucose results reached the recommended level, blood glucose control was considered acceptable. Blood glucose control was considered good if more than 90% reached the recommended level. If the pregnant women did not meet the recommended blood glucose targets, an insulin injection would be offered. If the insulin was rejected the women would be offered oral hypoglycaemics.

### Outcome measures and follow up

In this study, the postpartum glucose screening refers to 75-g, 2-h oral glucose tolerance test (OGTT) within 6 months postpartum based on the guidelines of the American Diabetes Association and the Chinese Medical Association [8, 9]. The World Health Organization [28] criteria for diabetes, impaired glucose tolerance and impaired fasting glucose were used to assess the postpartum glucose screening result: for diabetes: 2-h plasma glucose of OGTT ≥11.1 mmol/l or fasting plasma glucose ≥7.0 mmol/l; for impaired glucose tolerance: 2-h plasma glucose between 7.8 and 11.0 mmol/l; for impaired fasting glucose: fasting plasma glucose between 6.1 and 6.9 mmol/l.

An online postpartum glucose screening data sheet was used to collect the screening behaviour. The questions on the online postpartum glucose screening data sheet included asking if a postpartum glucose screening had been undertaken within 6 months postpartum (Yes or No), the name of the hospital and the date of the screening, and the methods used to detect glucose abnormality. Mothers were reminded to complete the data sheet by a mobile-phone message. If the mother did not reply, a follow–up phone call was made within 2 weeks. If a mother had the postpartum glucose screening in the study hospital, the results were retrieved from her medical record. If screening was done at other hospitals or clinics, the results were obtained from the mother.

### Statistical analysis

Descriptive statistics were used to present demographic and perinatal characteristics and the health beliefs. Some continuous variables such as age and health beliefs were categorised/dichotomised using clinical reference values or median values [29]. The mothers who had scores higher than the median on the 6 subscales were classified as having high level of health beliefs. Differences in rate of postpartum glucose screening among groups with different level of health beliefs, socio-demographic and perinatal characteristics were compared using the chi-squared test. Variables with *p* < 0.1 in the above tests were input into the multivariable logistic regression model to determine the predictors of the postpartum glucose screening. Maternal age and BMI were adjusted in multivariable analyses. Odds ratio (OR), adjusted odds ratio (AOR) and 95% confidence interval (95% CI) were calculated. Data analysis was performed using SPSS 22.0 for Windows (SPSS Inc., Chicago, IL, USA).

## Results

### Demographic and perinatal characteristics

**Fig. 1:**
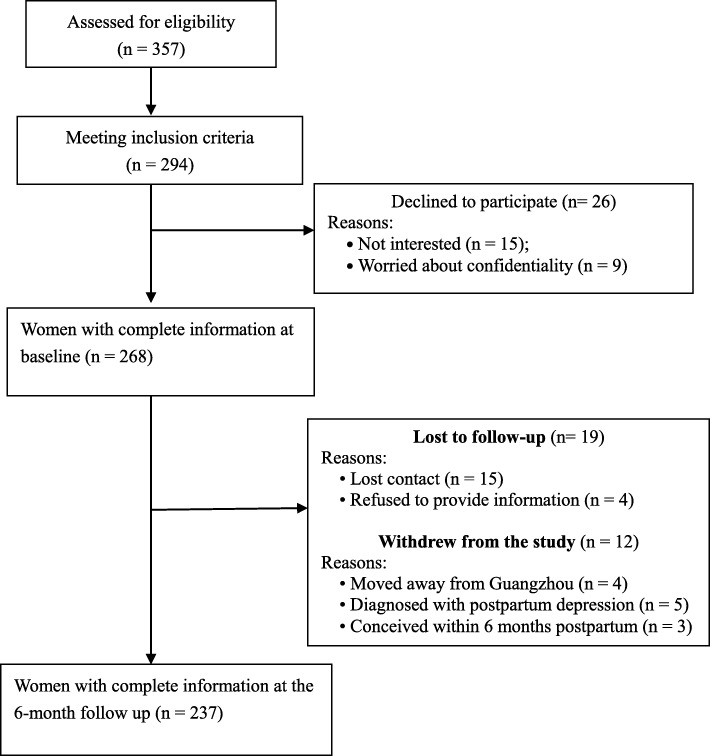
Flowchart of recruitment and loss to follow-up for the study

During the recruitment period, there were 357 mothers whose pregnancy was complicated with GDM. Of these, 294 met inclusion criteria. Twenty-six mothers (8.8%) refused to participate, 31 (10.5%) were lost to follow-up or withdrew from the study. At 6 months follow-up, 237 mothers finished the questionnaires (Fig. 1). There were no differences in socio-demographic characteristics between the mothers who completed the study and those who did not.

Table 1 presents the demographic characteristics of the mothers. The mean age of the mothers was 32.70 y (SD = 4.59, range = 22–44). Almost half of the mothers (45.6%) were college graduates or higher. More than one third of the mothers (35.9%) had a monthly household income > ¥ 9000 (US$1327), which was above the average monthly household income in Guangzhou [30] (Additional file 1).

**Table 1:** Demographic characteristics of the mothers (*n* = 237)

Characteristics	n	%
Ages (years)		
< 30	62	26.2
30–34	95	40.1
≥ 35	80	33.8
Education		
Senior high school or less	53	22.4
Junior college	76	32.1
College graduate	68	28.7
Graduate degree or above	40	16.9
Monthly family income		
< ¥5000 (about US$737)	37	15.6
¥5000–¥9000 (about US$737–US$1327)	115	48.5
> ¥9000 (aboutUS$1327)	85	35.9
Family history of diabetes		
No	164	69.2
Yes	59	24.9
Not known	14	5.9
Pre-pregnancy BMI (kg/m2)		
< 18.5	38	16.0
18.5–24.9	165	69.6
≥ 25.0	34	14.3

Table 2 presents the perinatal characteristics of mothers. In this study, half of the mothers (52.7%) had good glycaemic control during their pregnancy. However, only 7 mothers used insulin and 35 used oral agents to control blood glucose during pregnancy. Almost one fourth of the mothers (24.9%) had a family history of diabetes. More than half of the mothers (66.7%) exclusively breastfed their babies within the 6-month postpartum period. Childcare assistance was present for 62.9% of mothers.

**Table 2:** Perinatal characteristics of the mothers (n = 237)

Characteristics	n	%
Parity		
1	93	39.2
≥ 2	144	60.8
Gestational age when diagnosed with gestational diabetes mellitus (weeks)		
< 24	14	5.9
24–28	200	84.4
> 28	23	9.7
Gestational diabetes mellitus diagnostic OGTT glucose (mmol/l) (Mean ± S.D.)		
Fasting plasma glucose	4.59 ± 0.65	
1-h plasma glucose	9.90 ± 1.60	
2-h plasma glucose	9.07 ± 1.40	
Management of gestational diabetes mellitus		
Diet or physical activity	195	82.3
Oral agents	35	14.8
Insulin injection	7	3.0
Blood glucose control during pregnancy		
Acceptable or good	125	52.7
Not good	70	29.5
Data were unavailable	42	17.7
Gestational weight gain		
Less than the recommended	122	51.5
Within the range of the recommended	91	38.4
More than the recommended	24	10.1
Mode of delivery		
Spontaneous vaginal birth or vacuum	146	61.6
Caesarean section	91	38.4
Infant feeding method		
Exclusive breastfeeding	158	66.7
Mixed feeding	69	29.1
Formula feeding	10	4.2
Having someone help with childcare		
Yes	149	62.9
No	88	37.1

*OGTT* Oral glucose tolerance test

### Health beliefs

Table 3 presents the mean item score and the median score of the 6 subscales of the RC-CHBMS in descending order. The mothers had a high level of perceived benefits, self-efficacy and health motivation with the mean item score above 3.5 on these three subscale. Less than half of the mothers had a high level of perceived benefits (49.8%); self-efficacy (40.9%); health motivation (43.5%) and perceived barriers (48.9%). Only one third of the mothers (33.8%) had a high level of perceived seriousness of diabetes mellitus; while two thirds of the mothers had a high level of perceived susceptibility.

**Table 3:** The mothers’ scores on the subscales of the RC-CHBMS (n = 237)

Variables	Mean ± SD	Median	Number and percent > median (High)	Number and percent ≤ median (Low)
Perceived benefits	3.83 ± 0.29	3.83	118 (49.8%)	119 (50.2%)
Self-efficacy	3.75 ± 0.36	3.60	97 (40.9%)	140 (59.1%)
Health motivation	3.68 ± 0.32	3.80	103 (43.5%)	134 (56.5%)
Perceived seriousness	2.75 ± 0.37	2.83	80 (33.8%)	157 (66.2%)
Perceived susceptibility	2.63 ± 0.49	2.60	144 (60.8%)	93 (39.2%)
Perceived barriers	1.73 ± 0.35	1.67	116 (48.9%)	121 (51.1%)

### Postpartum glucose screening

Of the 237 mothers, 91 had postpartum glucose screening, which presents a postpartum glucose screening rate of 38.4%. Of the 91 mothers, 3 (3.3%) were classified with impaired fasting glucose, 24 (26.4%) with impaired glucose tolerance and 1 (1.1%) with diabetes. In addition, 24.5% (58 of the 237) mothers reported that they had monitored their finger stick capillary blood glucose irregularly at home.

Mothers were more likely to have postpartum glucose screening if they were first-time mothers [OR 2.153, 95% CI (1.257, 3.687)], or they had a family history of diabetes [OR 2.320, 95% CI (1.266, 4.250)] (Table 4). Mothers with high level of perceived benefits [OR 4.772, 95% CI (2.695, 8.451)], high level of perceived seriousness [OR 3.102, 95% CI (1.773, 5.430)], or high level of perceived susceptibility [OR 3.550, 95% CI (1.962, 6.422)] were more likely to undertake postpartum glucose screening. Blood glucose management during pregnancy was not related to postpartum glucose screening.

**Table 4:** Differences in rates of postpartum glucose screening between the mothers with high level of health beliefs and low level of health beliefs; and among the various socio-demographic and perinatal sub-groups (*n* = 237)

Characteristics	Postpartum Glucose screening		
	Yes (*n* = 91)	No (*n* = 146)	*OR (95% CI)*
Parity			
1	46 (49.5)	47 (50.5)	2.153 (1.257, 3.687)
≥ 2	45 (31.2)	99 (68.8)	Ref.
Family history of diabetes			
Yes	33 (55.9)	26 (44.1)	2.320 (1.266, 4.250)
No	58 (35.4)	106 (64.6)	Ref.
Not known	0 (0.0)	14 (100.0)	0.000
Mode of delivery			
Spontaneous vaginal birth or vacuum	63 (43.2)	83 (56.8)	1.708 (0.983, 2.968)^a^
Caesarean section	28 (30.8)	63 (69.2)	Ref.
Having someone help with childcare			
Yes	64 (43.0)	85 (57.0)	1.701 (0.974, 2.970)^a^
No	27 (20.7)	61 (69.3)	Ref.
Perceived benefits			
Low	25 (21.0)	94 (79.0)	Ref.
High	66 (55.9)	52 (44.1)	4.772 (2.695, 8.451)
Self-efficacy			
Low	52 (37.1)	88 (62.9)	Ref.
High	39 (40.2)	58 (59.8)	1.138 (0.669, 1.936)
Health motivation			
Low	46 (34.3)	88 (65.7)	Ref.
High	45 (43.7)	58 (56.3)	1.484 (0.875, 2.517)
Perceived seriousness			
Low	46 (29.3)	111 (70.7)	Ref.
High	45 (56.2)	35 (43.8)	3.102 (1.773, 5.430)
Perceived susceptibility			
Low	20 (21.5)	73 (78.5)	Ref.
High	71 (49.3)	73 (50.7)	3.550 (1.962, 6.422)
Perceived barriers			
Low	51 (42.1)	70 (57.9)	1.384 (0.818, 2.343)
High	40 (34.5)	76 (65.5)	Ref.

^a^*p* < 0.1

Variables that had significant correlation with postpartum glucose screening were retained in the logistic regression model. The best-fit regression model revealed 4 variables predicting postpartum glucose screening, including first-time mother [OR 2.618 (95% CI: 1.398–4.901)], high perceived susceptibility score [OR 2.173 (95% CI: 1.076–4.389)], high perceived seriousness score [OR 1.988 (95%CI: 1.020–3.875)] and high perceived benefits score [OR 2.978 (95%CI: 1.540–5.759)] (Table 5).

**Table 5:** Predictors of postpartum glucose screening within 6 months postpartum among Chinese mothers with a history of gestational diabetes mellitus (n = 237)

Variables	*B*	*wald*	*p*	*Adjusted OR*	*95%CI*	
					*Lower*	*Upper*
Parity^a^						
1	0.962	9.043	0.003^**^	2.618	1.398	4.901
≥ 2	Reference					
Perceived susceptibility	0.776	4.678	0.031^*^	2.173	1.076	4.389
Perceived seriousness	0.687	4.077	0.043^*^	1.988	1.020	3.875
Perceived benefits	1.091	10.517	0.001^**^	2.978	1.540	5.759

Adjusted for maternal age, pre-pregnancy body mass index, family history of diabetes, mode of delivery, childcare, perceived susceptibility, perceived seriousness and perceived benefits

^a^Parity: 1 = primipara, ≥ 2 = multipara

^*^p < 0.05, ^**^p < 0.01

## Discussion

According to our knowledge, this is the first study to explore the predictors of postpartum glucose screening among Chinese mothers with a history of GDM based on the Health Belief Model. The findings of the present study confirm the evidence that mothers with a history of GDM have a high risk for abnormal glucose regulation [2, 4, 31]. Therefore, all mothers with a history of GDM should have postpartum glucose screening. However, in the present study, only 91 (38.4%) of 237 mothers underwent this screening within 6 months postpartum, which was lower than the rates from Japan and Singapore. Kugishima et al. [11] reported that 65.7% of Japanese mothers with a history of GDM underwent postpartum glucose screening within 6 to 8 weeks postpartum. Suan [20] reported that 81.9% of Singaporean mothers with a history of GDM performed postpartum glucose screening within 6 to 8 weeks postpartum.

The lower rate of postpartum glucose screening in the present study may be related to the fact that mothers with a history of GDM were not advised to undertake postpartum glucose screening during pregnancy and were also not reminded to do so after discharge from the hospital. Currently, in mainland China healthcare professionals tend to assume that GDM disappears after delivery. Whilst occupied with caring for the baby they neglect to inform the pregnant women to screen for blood glucose abnormality after the birth [10]. It was only when a mother with GDM remained in the hospital after birth, that postpartum screening for diabetes by an obstetric nurse in the postnatal ward was recommended. Following discharge from hospital community nurses visited mothers with GDM twice within the first month postpartum, focusing on breastfeeding and the mothers’ physical recovery. Community nurses are not asked to remind these mothers to screen for diabetes as part of standard care [32].

Chang et al. [10] suggests that a reminder from health professionals is a key reason mothers with a history of GDM undertake postpartum glucose screening. A Cochrane literature review found that a reminder system for mothers with a history of GDM could increase the rate of postpartum glucose screening [33]. The findings of the present study indicate that there is an urgent need for postnatal healthcare professionals to remind mothers with a history of GDM to undertake postpartum glucose screening. A reminder system incorporating alerts into the mothers’ electronic medical records could be established, along with short text messages, emails or telephone calls as reminders.

Interestingly, there were 58 (24.5%) mothers with a history of GDM who monitored their finger stick capillary blood glucose irregularly by themselves. The finger test is however affected by temperature, humidity, operating practice and is not recommended to detect impaired fasting plasma and impaired glucose tolerance [34, 35]. The OGTT is more sensitive at 100% compared with 67% for the fasting plasma glucose test [34]. Both the Chinese Medical Association [9] and American Diabetes Association [8] recommend the OGTT as the standard method of postpartum screening for diabetes. Considering that the mothers in the present study had a high level of health motivation and perceived less barriers to diabetes screening, one explanation for this finding may be due to mothers’ lack of knowledge about the OGTT as the standard method of screening for diabetes. The findings of the present study suggested that healthcare professionals should not only remind the mothers to have postpartum glucose screening, but should also inform them that the OGTT is the standard method used to screen for glucose abnormality in the postpartum period.

The present study also found that parity predicted a woman’s motivation to attend for postpartum glucose screening. First-time mothers were more likely to screen for glucose abnormality than those who had more than one child. This was consistent with previous studies where mothers who had more children were less likely to screen for glucose abnormality in the postpartum period [19, 31, 35]. Mothers with only one baby have relatively more available time for postpartum glucose screening than those who have more than one child which may account for this [36, 37].

Concerning the Health Belief Model, mothers with a history of GDM who perceived higher susceptibility to diabetes, perceived seriousness of diabetes and higher benefits of postpartum screening for diabetes were more likely to undertake postpartum glucose screening. This finding is consistent with the Health Belief Model [15] and studies on breast cancer screening [38]. Chang et al. [10] further indicated that a woman’s belief that GDM would disappear after delivery was the second highest occurring reason for not performing postpartum glucose screening.

Mothers with a history of GDM have a moderate level of perceived susceptibility and perceived seriousness to diabetes and may not realize the risks subsequent diabetes. This may be another reason why many mothers with a history of GDM in the present study did not undertake postpartum glucose screening. This finding was consistent with a previous study, in which 74% of Australian mothers with a history of GDM did not perceive themselves at high or very high risk for developing diabetes in the future [39]. The findings of the present study suggested that an education program with a strong focus on perceived susceptibility and perceived seriousness to diabetes and the benefits of postpartum glucose screening may improve the uptake of such screening among the mothers with a history of GDM.

In relation to the management of their GDM, the present study found that only a small percent of pregnant women managed their blood glucose by insulin injection or oral medicine. Almost one third of the mothers did not effectively manage blood glucose during pregnancy. Only 3% of the mothers (*n* = 7) managed their blood glucose through the injection of insulin and 14.8% (*n* = 35) by oral medicine. This may be because most Chinese pregnant women with GDM are resistant when treatment with insulin injection or oral agents is advised [40]. Insulin injection is perceived as inconvenient and women fear the side effects of oral agents on the foetus. Moreover, some of them may mistakenly believe that they have to rely on insulin on an ongoing basis once it has been used initially [40]. The findings of the present study are consistent with a previous study which suggested that health care professionals should put more effort into helping pregnant women with a history of GDM to understand how to manage GDM correctly, especially regarding insulin injection and oral medicine [41].

This study had some limitations. The study was conducted in one hospital. The majority of mothers were middle class women with a high-level of education [30]. Our findings may not be transferable to other settings or to women from a different social class, with lower levels of education or to rural women.

## Conclusion

This study has significant clinical utility for health care professionals working with Chinese mothers with a history of GDM. To identify mothers who may not undergo postpartum glucose screening, health care professionals are advised to assess a mother’s parity, health beliefs in relation to perceived susceptibility, perceived seriousness and perceived benefits. Health care professionals could develop strategies following assessment to encourage these mothers to undergo postpartum glucose screening. These strategies for example could be establishing a reminder system and providing education focused on the risk and seriousness of GDM for diabetes and the benefits of postpartum glucose screening.

## Additional file

Additional file 1: The socio-demographic data sheet of the participants. This data sheet includes the socio-demographic information of all the participants. (SAV 3 kb)

## Abbreviations

BMI: Body Mass Index
GDM: Gestational diabetes mellitus
OGTT: Oral glucose tolerance test
